# Supplementary material for: A SEVA-based, CRISPR-Cas3-assisted genome engineering approach for Pseudomonas with efficient vector curing
Source: Microbiol Spectr. 2023 Nov 17;11(6):e02707-23. doi: 10.1128/spectrum.02707-23 (PMC10715078; doi:10.1128/spectrum.02707-23)
Supplement: Supplemental Figures and Tables — Supplemental Tables with primers sequences, sequences of genetic parts and vector details. Supplemental figures with PCR controls of engineered strains, details of SNP analysis upon whole genome sequencing of selected strains and vector maps of the main plasmids. [file spectrum.02707-23-s0001.docx]

## Supporting information

### Supplementary Tables

Table S1: All primers used in this work. Red: overhang for ligation in pCas3cRh with BsaI; yellow: BsaI recognition site; blue: SapI recognition site; grey: BsaI/SapI restriction site; green: U nucleotide for USER cloning

| **Name** | **Sequence** |
| --- | --- |
| *Vector construction – pSEVA52-oriT* | |
| oriT_spacer_F | GAAACGCACGATATACAGGATTTTGCCAAAGGGTTCGTGG |
| oriT_spacer_R | GCGACCACGAACCCTTTGGCAAAATCCTGTATATCGTGCG |
| oriTcas3_SEVA_F | GACGGTCTCCCTCTACTAGTCTTGGACTCCTG |
| oriTcas3_SEVA_R | GTCGGTCTCCGGTGTTAATTAAAGGCATCAAATAAAACG |
| oriT_cas3_F | GACGGTCTCCCACCACAATTCAGCAAATTGTGAAC |
| oriT_cas3_R | GTCGGTCTCCAGAGCACTAGGTTTCAATCCAC |
| *Vector assembly – pCas3cRh with spacers* | |
| pCas3cRh_F | CAGGAAATGCGGTGAGC |
| pCas3cRh_R | GAGCAGCTAATTCACCGC |
| PP5388_spacer_F | GAAACAGATCATGGTAACCCCGGCCGCTGGAGCCATTTCG |
| PP5388_spacer_R | GCGACGAAATGGCTCCAGCGGCCGGGGTTACCATGATCTG |
| FleS_spacer_F | GAAACAACCAGATGTCCAGCCAGCTCAGCGAGTCCTACAG |
| FleS_spacer_R | GCGACTGTAGGACTCGCTGAGCTGGCTGGACATCTGGTTG |
| PrpL_spacer_F | GAAACTACAACACCACCCAGTGCTACGGCGACGCCTCGAG |
| PrpL_spacer_R | GCGACTCGAGGCGTCGCCGTAGCACTGGGTGGTGTTGTAG |
| PA2560_spacer_F | GAAACGCCTGCTGGACGAGGTGCAATACCCGGATGCGCCG |
| PA2560_spacer_R | GCGACGGCGCATCCGGGTATTGCACCTCGTCCAGCAGGCG |
| *Vector assembly – HDR templates in pSEVAX31* | |
| SEVA_PS1 | AGGGCGGCGGATTTGTCC |
| SEVA_PS2 | GCGGCAACCGAGCGTTC |
| pSNW_seq_F | TGTAAAACGACGGCCAGT |
| pSNW_seq_R | CTTTACACTTTATGCTTCCGG |
| pSEVA231 _F | GACGGTCTCCAGTCACTAGTCTTGGACTCCTG |
| pSEVA231 _R | GTCGGTCTCCCTAGTTAATTAAAGGCATCAAATAAAACG |
| pSEVA131_FleS_F | TACGGTCTCAAGTCACTAGTCTTGGACTCCTG |
| pSEVA131_Fles_R | GGGGGTCTCCGGACTTAATTAAAGGCATCAAATAAAACG |
| pSEVA131_PrpL_F | GACGGTCTCCGGGCACTAGTCTTGGACTCCTG |
| pSEVA131_PrpL_R | GTCGGTCTCCTTACTTAATTAAAGGCATCAAATAAAACG |
| pSEVA131_PA2560_F | GACGGTCTCCTGGCACTAGTCTTGGACTCCTG |
| pSEVA131_PA2560_R | GTCGGTCTCCGAGCTTAATTAAAGGCATCAAATAAAACG |
| pSNW2_F | ATAGGTCTCAAGTCGACCTGCAGGCATGCAAGC |
| pSNW2_R | ATAGGTCTCACTAGAGGATCCCCGGGTACCGAGC |
| PP5388_HAup_F | ATAGGTCTCACTAGTGACCGCACCGTTGCTCTCTCTCTTCG |
| PP5388_HAup_R | ATAGGTCTCATAGATGAATCGCCCTGACTTC |
| PP5388_HAdn_F | ATTGGTCTCAGGCTGATCATGGTAACCCCGGCCGC |
| PP5388_HAdn_R | ATAGGTCTCAGACTGCCCTGGTCGACGATATTG |
| Trrnb_F | ATAGGTCTCATCTAATTTGTCCTACTCAGGAGAGCG |
| T0term_R | CTTGGTCTCTAGCCCTGGATTCTCACCAATAAAAAAC |
| FleS_HAup_F | TTAGGTCTCCGTCCGCGAACTGGCGAACC |
| FleS_HAup_R | TATGGTCTCCTTGCGTTTCTCTCGGTCGGCGCGACG |
| FleS_HAdn_F | ATAGGTCTCCAACGCACCCCATGGCAGCCAAAGTCC |
| FleS_HAdn_R | AACGGTCTCTGACTTCCTTGCCGGTCCCGGACTC |
| PrpL_HAup_F | GACGGTCTCCGTAATGGTAAGCGGCCAG |
| PrpL_HAup_R | GTCGGTCTCCCCGAATCGACTCCTTCAG |
| PrpL_HAdn_F | GACGGTCTCCTCGGCCAGCCAGGCTATCG |
| PrpL_HAdn_R | GTCGGTCTCCGCCCGCGAGCATTCCTCCTG |
| PA2560_HAup_F | GACGGTCTCCGCTCTCCAGTATTTCGTC |
| PA2560_HAup_R | GTCGGTCTCCGCGAGTCAGATCCTTCG |
| PA2560_HAdn_F | GACGGTCTCCTCGCCGACGCCAGGAGCAC |
| PA2560_HAdn_R | GTCGGTCTCCGCCAAGGCGCAAGCCTG |
| *Verification of genomic modifications* | |
| PP5388_up | ATTCTTTGGAGCAACGGAAC |
| PP5388_dn | CAGCTGCAAGTGGAGATC |
| FleS_up | AGAAGATGATCGAGGAC |
| FleS_dn | GAACCTTTCTCGTGGC |
| PrpL_up | CCACGTCAGCGGCAAG |
| PrpL_dn | GGCTCTGCCGTTTCAC |
| PA2560_up | GTTGCCTGCCTTGTC |
| PA2560_dn | GACGCCTTGCTTCTC |
| *Vector construction – pCas3-XX and pSEVAX3-GG vector sets* | |
| pCas3_Ab_F | AACAGGAGUAGCGTCAGACCCCGTAGAAAAG |
| pCas3_Ab_R | ATCCATCCUGCGTGAGCGCATACGCTACTTG |
| Ab_F | ACTCCTGTUGATAGATCCAGTAATGACCT |
| Ab_R | AGGATGGAUATACCGAAAAAATCGCTATAA |
| pSX31_GG_F | AGGTCTCTGAAUCCCCGGGTACCGAGC |
| pSX31_GG_R | AGTGGTCTCGUCGCCCGGCAAAACCGGGCGTGGATTGAAACGCTTGCGGCCGCGTCG |
| P14g-BCD2-GFP_F | ATTCAGAGACCUGCCCATTGACAAGGC |
| P14g-BCD2-GFP_R | ACGAGACCACUAGTCATTTATTTGTAGAG |
| pSX31_Ab_F/R | ATCCATCCUTTTTCGCACGATATACAGG |
| pSX31_Ab_F/R | AACAGGAGUCCAAGACTAGTCGC |
| ApR_BsaI_F | AGCCAAUCGACTGGCGAGCGGCATC |
| ApR_BsaI_R | ATTGGCUAAGACCGCGGTCCGCGCGT |

Table S2: All vectors used in this work.

| **Name** | **Relevant features** | **Reference** |
| --- | --- | --- |
| pCas3cRh | *oriT*; *oriV(**pRO1600/ColE1)*; RhaRS/*PrhaBAD::gRNA-cas3-cas5-cas8-cas7*; MCS; Gm^R^ | (1) |
| pCas3cRh-PP_5388 | pCas3cRh derivative with a PP5388-targeting spacer | This work |
| pCas3cRh-FleS | pCas3cRh derivative with a FleS-targeting spacer | This work |
| pCas3cRh-PrpL | pCas3cRh derivative with a PrpL-targeting spacer | This work |
| pCas3cRh-PA2560 | pCas3cRh derivative with a PA2560-targeting spacer | This work |
| pCas3cRh-oriT | pCas3cRh derivative with an oriT-targeting spacer | This work |
| pCas3-Amp | *oriT*; *oriV(pRO1600/ColE1)*; RhaRS/*PrhaBAD::gRNA-cas3-cas5-cas8-cas7*; MCS; Amp^R^ | This work |
| pCas3-Km | *oriT*; *oriV(pRO1600/ColE1)*; RhaRS/*PrhaBAD::gRNA-cas3-cas5-cas8-cas7*; MCS; Km^R^ | This work |
| pCas3-Sm | *oriT*; *oriV(pRO1600/ColE1)*; RhaRS/*PrhaBAD::gRNA-cas3-cas5-cas8-cas7*; MCS; Sm^R^ | This work |
| pCas3-Gm | *oriT*; *oriV(pRO1600/ColE1)*; RhaRS/*PrhaBAD::gRNA-cas3-cas5-cas8-cas7*; MCS; Gm^R^ | This work |
| pCas3-Apr | *oriT*; *oriV(pRO1600/ColE1)*; RhaRS/*PrhaBAD::gRNA-cas3-cas5-cas8-cas7*; MCS; Apr^R^ | This work |
| pSEVA231 | SEVA vector, *oriT*; *oriV(pBBR1)*; MCS; Km^R^ | (2) |
| pSEVA23-PP_5388 | pSEVA231 derivative with PP5388(HA1)-T1-*p14c-BCD22-phi15lys(G3RQ)-*T0-PP0013(HA2) | This work |
| pSNW2 | Integration vector; *oriT*; *oriV(R6K)*; *P_14g_-BCD2-msfGFP* selection marker; MCS*,* Km^R^ | (3) |
| pSNW2-PP_5388 | pSNW2 with PP5388(HA1)-T1-*p14c-BCD22-phi15lys(G3RQ)-*T0-PP0013(HA2) | This work |
| pSEVA131 | SEVA vector, *oriT*; *oriV(pBBR1)*; MCS; Amp^R^/Cb^R^ | (2) |
| pSEVA13-FleS | pSEVA131 derivative with FleS(HA1)-FleS(HA2) | This work |
| pSEVA13-PrpL | pSEVA131 derivative with PrpL(HA1)-PrpL(HA2) | This work |
| pSEVA13-PA2560 | pSEVA131 derivative with PA2560(HA1)-PA2560(HA2) | This work |
| pSEVA521 | SEVA vector, *oriT*; *oriV(RK2)*; MCS; Tc^R^ | (2) |
| pSEVA52-oriT | pSEVA521 derivative with *PrhaBAD::CRISPRrepeat-oriTspacer-CRISPRrepeat* | This work |
| pSEVA13-GG | SEVA vector, *oriT*; *oriV(pBBR1)*; GoldenGate compatible with BsaI sites flanking a *P_14g_-BCD2-msfgfp* reporter cassette; Amp^R^/Cb^R^ | This work |
| pSEVA23-GG | SEVA vector, *oriT*; *oriV(pBBR1)*; GoldenGate compatible with BsaI sites flanking a *P_14g_-BCD2-msfgfp* reporter cassette; Sm^R^/Sp^R^ | This work |
| pSEVA43-GG | SEVA vector, *oriT*; *oriV(pBBR1)*; GoldenGate compatible with BsaI sites flanking a *P_14g_-BCD2-msfgfp* reporter cassette; Km^R^ | This work |
| pSEVA63-GG | SEVA vector, *oriT*; *oriV(pBBR1)*; GoldenGate compatible with BsaI sites flanking a *P_14g_-BCD2-msfgfp* reporter cassette; Gm^R^ | This work |
| pSEVA83-GG | SEVA vector, *oriT*; *oriV(pBBR1)*; GoldenGate compatible with BsaI sites flanking a *P_14g_-BCD2-msfgfp* reporter cassette; Apr^R^ | This work |
| pBG42 | pBG derivative with P14g-BCD2-msfGFP | (4) |

Table S3: All strains used in this work.

| **Name** | **Description** | **Reference** |
| --- | --- | --- |
| *E. coli* TOP10 | Intermediate host for vector cloning (Invitrogen^TM^); F- *mcrA* Δ( *mrr-hsd*RMS-*mcr*BC) Φ80*lac*ZΔM15 Δ *lac*X74 *rec*A1*ara*D139 Δ( *araleu*)7697 *gal*U *gal*K *rps*L (StrR) *end*A1 *nup*G | - |
| *P. putida* KT2440 | Derivative of *P. putida* mt-2 lacking the TOL plasmid | (5) |
| *P. putida* SEM11 | Genome reduced derivative of *P. putida* KT2440 with deletion of prophages, ꞵ-lactamases, *benABCD* and *pvdD* | (6) |
| *P. putida* KT-phi15lys | Derivative of *P. putida* KT2440 with a T1-p14c-BCD22-phi15lys(G3RQ)-T0 integration in the PP5388 locus | This work |
| *P. putida* S-phi15lys | Derivative of *P. putida* SEM11 with a T1-p14c-BCD22-phi15lys(G3RQ)-T0 integration in the PP5388 locus | This work |
| *P. aeruginosa* PAO1 |  | (7) |
| *P. aeruginosa* PAO1 Δ*fleS* | Derivative of *P. aeruginosa* PAO1 with a deletion of the *fleS* gene | This work |
| *P. aeruginosa* PAO1 Δ*prpL* | Derivative of *P. aeruginosa* PAO1 with a deletion of the *prpL* gene | This work |
| *P. aeruginosa* PAO1 *Δpa2560* | Derivative of *P. aeruginosa* PAO1 with a deletion of the *pa2560* gene | This work |

Table S4: All sequences of homology arms and inserts used in this work. HA: homology arm, up: upstream, dn: downstream

| **Name** | **Sequence** |
| --- | --- |
| PP_5388 HA up | TGACCGCACCGTTGCTCTCTCTCTTCGTGATTCCCGCAGCGTATTGGCTGGTCCGACGCCGCGATCTTGTAGTACCTCATAATTCCACACCAGGAGACATCCGATGAAAAAGCTCTACCTCAGCATTGCACTGCTCTTTGCCTTCGCATCAGGCGCGCAAGCCCAAGACTCCATGGCCGGGATGAGCATGGATGGAATGGATATGAAGGAAACCCAATCAGCACCTTCCGCTCATGCAGAAGGGACGGTAAAGGCAATTGACGCCCAAGGCGGCACAGTGACCCTGATGCATGGACCGGTTGCTGCGTTGAAATGGCCGGCCATGACCATGGCCTTCAAAGCCTCTGCGCAACAGCTCGATGGATTGAAGGTGGGAGACAACGTAGAGTTTGATTTCCGGATGGATGGCAGCACGGCAACGATTGTTGATATTCGCAAACAGTAATTCGCCTAATTATCCTGTCTGCCAACGCTTGATTCGCCCATGACCTCCTGACGCCTCCTAGCAACTGGAAAAGCTTGGAAGCATCTAGGAAGTCAGGGCGATTCA |
| PP_5388 HA dn | GATCATGGTAACCCCGGCCGCTGGAGCCATTTCTGAAGTACTGACACGGCGCTGAAGCGATGTGGCAGTTCCGCCTAAGGCGGCGAGCAGCAGCGAGAAAAGATCGTCACCCCAAGTTTTCTTCTTGCAGGCGACTGAATAGAGGAGAAATCGAGATAACAGCTCGCAAGGCATCCTGATTGGCGCCCGACCATGCCTGATCCTGTACATCATTGAGCACGTGCTGTGTCGGAATTGGCCGAAACGCTTGCGTCGCGGGGTCGTAGACGTAAACGTGAAGACCTATTTCAATCGCTCGCGAGGCCTGACGGTGCCAGTTAAAGTTGCCACCATTCGTCATCGTGTCCGCAGCTACGAGGATGTCGTAGTCATTAACGATGTAGCCGAAAAGTACATGCTGCGAGATATCGCGGAGAGCTAGAGAATGACGCCTGCTCGCCGATCGCCAGACTAGGTTGCGTGCAAAGTGTCGCCTACTGACCCTTGGGTCATCCAAAAGCGTGACCTTGTCGTAATAGGCTACTGACAAATCAATATCGTCGACCAGGGC |
| *P14c-BCD22-phi15lys(G3RQ)* cassette | ATTTGTCCTACTCAGGAGAGCGTTCACCGACAAACAACAGATAAAACGAAAGGCCCAGTCTTTCGACTGAGCCTTTCGTTTTATTTGATGCCTTTAATTAATCTAGTGAATTGACATGTCAATTTTTATGTTGTATAATATAACTAGCAGGCCCAAGTTCACTTAAAAAGGAGATCAACAATGAAAGCAATTTTCGTACTGAAACATCTTAATCATGCCTAGGAAGTTTTCTAATGGCTCGTCAAGTCAAATTTAAGGAGCGCTTGAGTACCAAGATGATCGTTGTGCACTGTTCGGCCACCAAGGCCAGCATGGACATCGGTCGTAAAGAGATCCAAATGTGGCACGTTCAGCAAGGCTGGCTGGCCATTGGGTACCACCTCGTGATTCGTCGCGACGGTACCATCGAGCAAGGCCGACCACACAAGGCCATCGGGTCCCACGTTAAGGGTCACAACAGTGACTCCATCGGGATCTGCCTCGTGGGCGGTATCGACGACTCGGGGAAACCTGAGGACAACTTCACGGACCAGCAAAAGGCTGCGCTGAGCGGCCTGCTGTGGGACATGACGCAATCTGGCGTGACCTATGGGGACACCTATAAGGAGCTGCCAGTGGTTGGTCACCGTGATCTCGATAGTGGTAAAGCCTGCCCGAGCTTCGATGTGAAGGCATGGTGGGCCGCTCAGATCAATTAACACTAGTCTTGGACTCCTGTTGATAGATCCAGTAATGACCTCAGAACTCCATCTGGATTTGTTCAGAACGCTCGGTTGCCGCCGGGCGTTTTTTATTGGTGAGAAT |
| FleS HA up | CCGCGAACTGGCGAACCTGGTGGAGCGCCTGGCGATCATGCATCCCTACGGGGTGATCGGGGTCGGCGAACTGCCGAAGAAATTCCGCCATGTCGACGACGAGGACGAGCAACTCGCCAGCAGCCTGCGCGAAGAGCTGGAAGAGCGCGCGGCGATCAACGCCGGGCTGCCGGGAATGGACGCGCCGGCGATGCTGCCGGCCGAAGGCCTGGACCTCAAGGACTACCTGGCCAACCTCGAGCAGGGCCTGATCCAGCAGGCCCTCGACGACGCCGGCGGAGTGGTCGCGCGGGCCGCCGAACGCCTGCGCATCCGCCGCACCACGCTGGTAGAGAAGATGCGCAAGTACGGCATGAGCCGGCGTGACGACGACCTGTCGGATGATTGACAGGTCGTTTCGCAACGCTTTGATTTTCAAATGAAAAAAATTTAGGCACGGGTATTGCTATATCTCCGTCGACCGACAGAACCATGACGTCGCGCCGACCGAGAGAAACG |
| FleS HA dn | ACCCCATGGCAGCCAAAGTCCTGCTGGTCGAAGACGACCGCGCACTACGCGAAGCCCTCAGCGACACCCTGCTGCTGGGCGGTCACGAGTTCGTCGCCGTGGACTCGGCGGAGGCGGCGCTGCCGGTCCTGGCCCGCGAAGCCTTCAGCCTGGTGATCAGCGACGTGAACATGCCGGGCATGGACGGACACCAGTTGCTCGGCCTGATCCGTACACGCTACCCGCACCTGCCGGTGTTGCTGATGACCGCCTACGGCGCGGTCGATCGCGCCGTCGAGGCGATGCGCCAGGGCGCCGCCGACTACCTGGTCAAGCCGTTCGAGGCGCGGGCGCTGCTCGACCTGGTGGCGCGCCATGCGCTGGGCCAGTTGCCGGGCAGCGAGGAGGATGGTCCGGTGGCCCTGGAGCCGGCCAGCCGGCAGTTGCTGGAACTGGCCGCGCGGGTCGCGCGCAGCGATTCCACCGTGCTGATCTCCGGCGAGTCCGGGACCGGCAAGGAAG |
| PrpL HA up | GTAATGGTAAGCGGCCAGGGCTCTATATATGCCCCGCGATTATAGAGATGACTTATACGACCATAAGCCGGATATCCTGGAATTCAACCAAGCCAACATCGGAACGGTTTCCGAGCGGTGAATGGAAGATTCACCCAGCCTTATATTCGGCAGTGGTCAAACCGGATAACTTGAAAATCAGGATTTCGTTAACCCGATCACATCGACAGCTGCACCAGTAAAGCACTCATGCACTACATCCTGCACCTCTCTGAAGCAAACCGAAGGCTCTGCAGAGCCACTCCAGACCAAACTTTAGTTGGTAGAGAGAGCAATCCAACATCAATGGCAAGTGAAGGAAATAGCTATCTATTCTGCTATCCACAAACGCTTCTTCATATTTAATAGCTAGCAGAAACGAATATAAAAAATATTTGAACGACTCCGTCACACCTGCATATCTTTCGCCACGCGAACGATTGAGGAAGTTGCCCTCCCAAAAAACTGAAGGAGTCGATTC |
| PrpL HA dn | GGCCAGCCAGGCTATCGATCGCGCACCGGCGGATAACCGCGCAGCGGTTATTCGCCCTACGCCCGGATTGGCGCCTGGAAGTGCGGCTTCTTTCGTCGTCCGGGACAGGTAGGGCGCATAACGCCAACGGCGTTATCCGCCGTCTATCCCGCGCAATGGTTATTCGCCCCACGCCCGGATTGGCACCTGGAAGTGCGGCTTCTTTCGTCGTCCGGGACAGGTAGGGCGCATAACGCCAACGGTGTTATCCGCCGTTTATCCCGCGCAATGGTTATTCGCCCCACGCCTGGATCGGCGCGTGGAAGAGGGGCGTTATGCGCCGCCGTACGTTCAGTCCTGGCGGCTGGTGACTTCCAGCAGGTGGTAGCCGAACTGGGTCTTCACCGGCCCCTGGACCACGTTCAGCGGCGCGCTGAAGACCACCTGGTCGAACTCGCGGACCATCTGGCCGGGGCCGAACGAGCCCAGGTTGCCGCCGTCGCGGCCGGAGGGGCAGGAGGAATGCTCGCGGGC |
| PA_2560 up | GAGTCAGATCCTTCGACTGGCACAATGTGACATTCGTCACAGAGATTAACCGAAGCCCCGCAGAACATCCATGCCAGCTATTTCCCTTCGTCGGCTCTGGCCCGGCCCCTGCGTCGTCCTCAAGTTGTCAGATCCGCTGTCGATACTCTGCGAGCCGGAACACGCGCCATGCCCGCCTCGCCGGGCCACAGGGACGTCCTCGGCTGCCTCGTCGCAGCCTGCGTGCCGGTCCAACCTGGCAATCCATCGAGGCGTTCCATGCTGCAACAATCCCTACGTGCGCAAATCCTTGTCCTGCTCGGCGGCAGCCTGGCGGCGCTGCTACTCATAGCCCTGGCCTGCTTCGGCTCGCTGACCGGCGACGTACGCGCCTACCGCGAGCTGCTCGGCGGCCCCGTGCGGGCGGCGCAACTGATCGACGAGGCCAACCTGCAATTCCGCGGCCAGGTCCAGGAATGGAAGAACGTCCTGCTGCGCGGACGCCAGACGGAGGCCCAGACGAAATACTGGA |
| PA_2560 dn | GCCAAGGCGCAAGCCTGAACAAGCTAGCCGACTTGGCGAGGAACGCACAAATTTCATCGGCTGGCGTACAGGTATGGGACATCGGTACCAATAGCGAAGTCGCGCAATCCGTCAGTTTTTTTTGAATTTTTGCCATTGGAAAAAGTCTGTTCAGCGCAGCGTTTTTCCAAGTCCCATCCGGCCCGCAAGGAGAACCCATGGCTGAACCCCAGGACAAGTACACCCGGCGCACAGGCAGGACCTGGGCGGACGACCAGGCGACCTACAACCGTCTGCGCGAAGAAGCCGACGCCGCTCGCCAGAAGCTGCGCGAAAGCGGCTACAGCGGCGCCGAGTACGACCAGTTGCGTCAAGCCGCCTTCGATCTCAACCGCAAGGCCAACCAGTACTGGGAGCAGATGCTCAGCGACCTGCGCCAGGAAGACTGATCAGCGCCACGCCGGGAGACTGCCGCTCTCGGCCCGCACACCCCCTCCACTCTTTGCGTGCTCCTGGCGTCGGC |

Table S5: SNP analysis of P. putida KT-phi15lys, replicate 1. The nucleotide position of the integration cassette is 6 143 866 – 6 144 687.

| **Reference** | **Nucleotide position** | **Type** | **Reference** | **Alteration** | **Evidence** |
| --- | --- | --- | --- | --- | --- |
| genome | 533 982 | snp | A | G | G:31 A:0 |
| genome | 1 126 645 | ins | A | AC | AC:21 A:0 |
| genome | 1 720 346 | snp | C | T | T:27 C:0 |
| genome | 4 586 056 | ins | A | AC | AC:19 A:0 |

Table S6: SNP analysis of P. putida KT-phi15lys, replicate 2. The nucleotide position of the integration cassette is 6 143 866 – 6 144 687.

| **Reference** | **Nucleotide position** | **Type** | **Reference** | **Alteration** | **Evidence** |
| --- | --- | --- | --- | --- | --- |
| genome | 533 982 | snp | A | G | G:52 A:0 |
| genome | 1 126 645 | ins | A | AC | AC:20 A:0 |
| genome | 1 720 346 | snp | C | T | T:37 C:0 |
| genome | 4 586 030 | complex | CTGC | TCGCG | TCGCG:11 CTGC:0 |

Table S7: SNP analysis of P. putida S-phi15lys, replicate 1. The nucleotide position of the integration cassette is 5 786 593 – 5 787 404.

| **Reference** | **Nucleotide position** | **Type** | **Reference** | **Alteration** | **Evidence** |
| --- | --- | --- | --- | --- | --- |
| genome | 1 146 407 | snp | A | C | C:10 A:0 |
| genome | 5 200 648 | snp | T | C | C:14 T:0 |

Table S8: SNP analysis of P. putida S-phi15lys, replicate 2. The nucleotide position of the integration cassette is 5 786 593 – 5 787 404.

| **Reference** | **Nucleotide position** | **Type** | **Reference** | **Alteration** | **Evidence** |
| --- | --- | --- | --- | --- | --- |
| genome | 1 146 407 | snp | A | C | C:12 A:0 |
| genome | 5 200 648 | snp | T | C | C:17 T:0 |

### Supplementary Figures


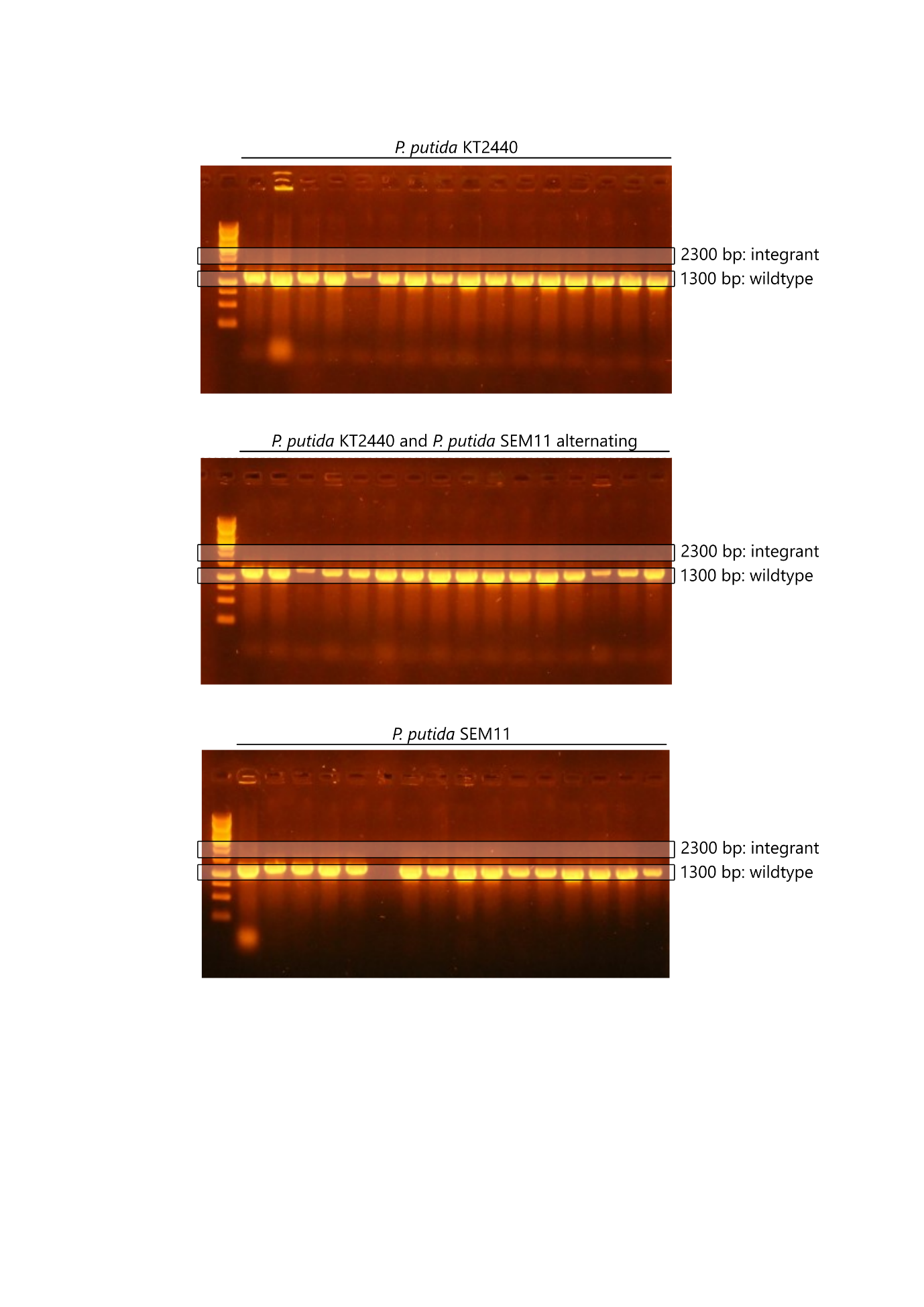


Figure S1: Integration of P_14c_-BCD22-phi15lys(G3RQ) in the PP_5388 locus in P. putida KT2440 and P. putida SEM11 with Cas3-based engineering. A PCR screen was performed on co-transformants (no induction) with genome-binding primers located outside of the homology arms. The first lane on each gel contains the commercial 1 kb GeneRuler (Thermo Scientific). Expected amplicon length of wildtype colonies and integrants are indicated on each gel.


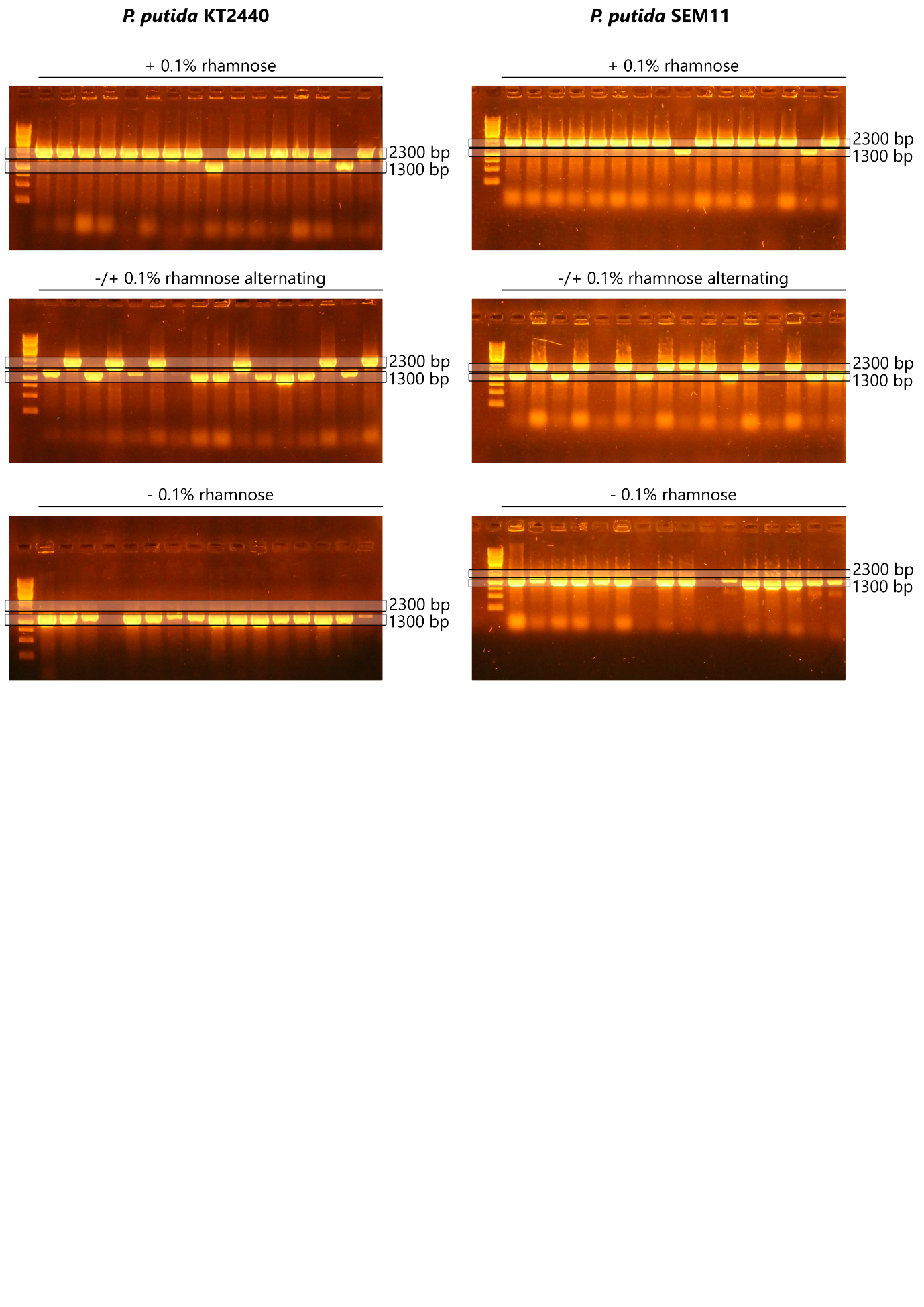


Figure S2: Integration of P_14c_-BCD22-phi15lys(G3RQ) in the PP_5388 locus in P. putida KT2440 (left) and P. putida SEM11 (right) with Cas3-based engineering. A PCR screen was performed on co-transformants (with and without overnight induction with 0.1% rhamnose) with genome-binding primers located outside of the homology arms. The first lane on each gel contains the commercial 1 kb GeneRuler (Thermo Scientific). Expected amplicon length of wildtype colonies (1300 bp) and integrants (2300 bp) are indicated on each gel.


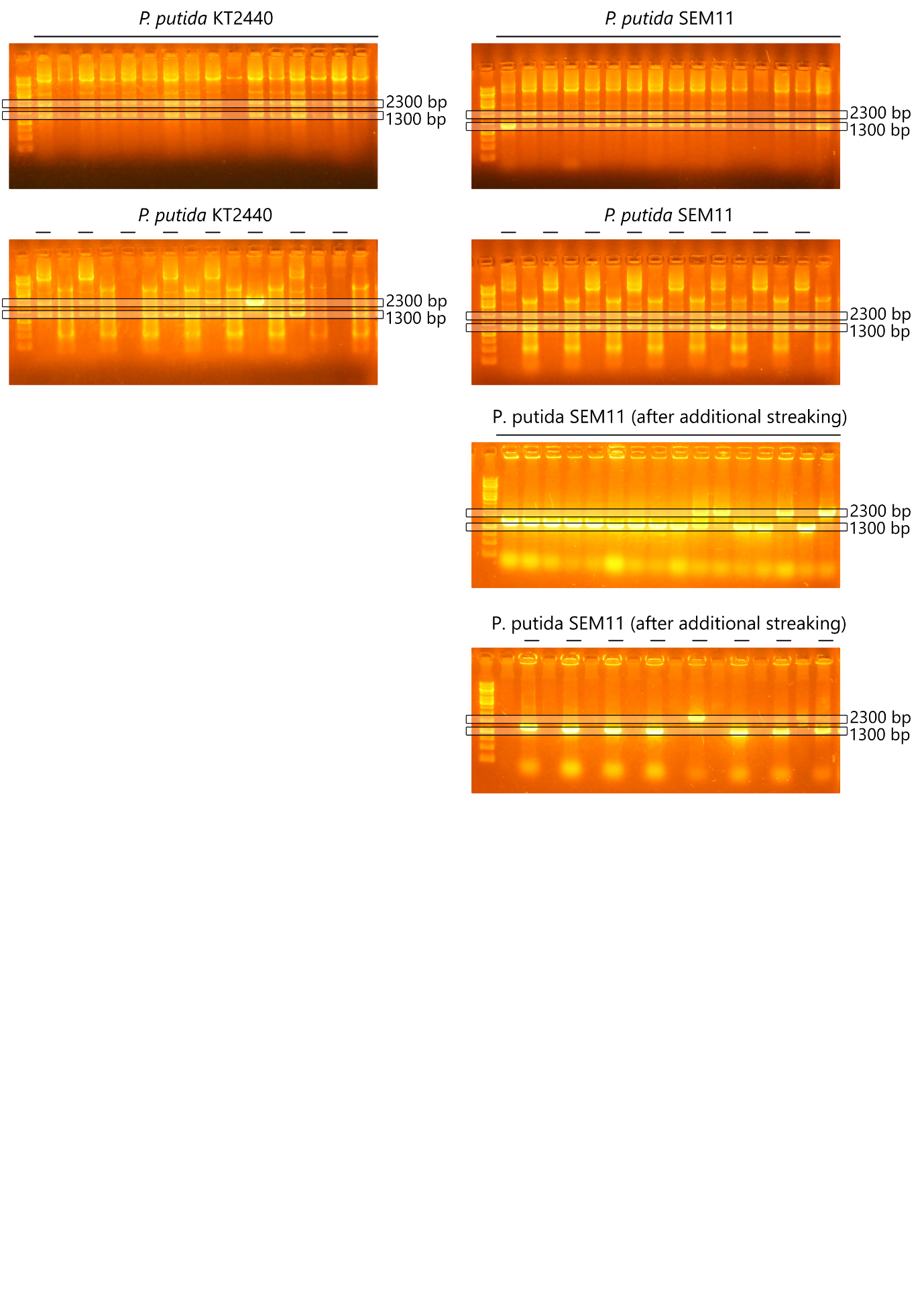


Figure S3: Integration of P_14c_-BCD22-phi15lys(G3RQ) in the PP_5388 locus in P. putida KT2440 (left) and P. putida SEM11 (right) with homologous recombination. A PCR screen was performed on co-transformants with genome-binding primers located outside of the homology arms. The first lane on each gel contains the commercial 1 kb GeneRuler (Thermo Scientific). Expected amplicon length of wildtype colonies (1300 bp) and integrants (2300 bp) are indicated on each gel.


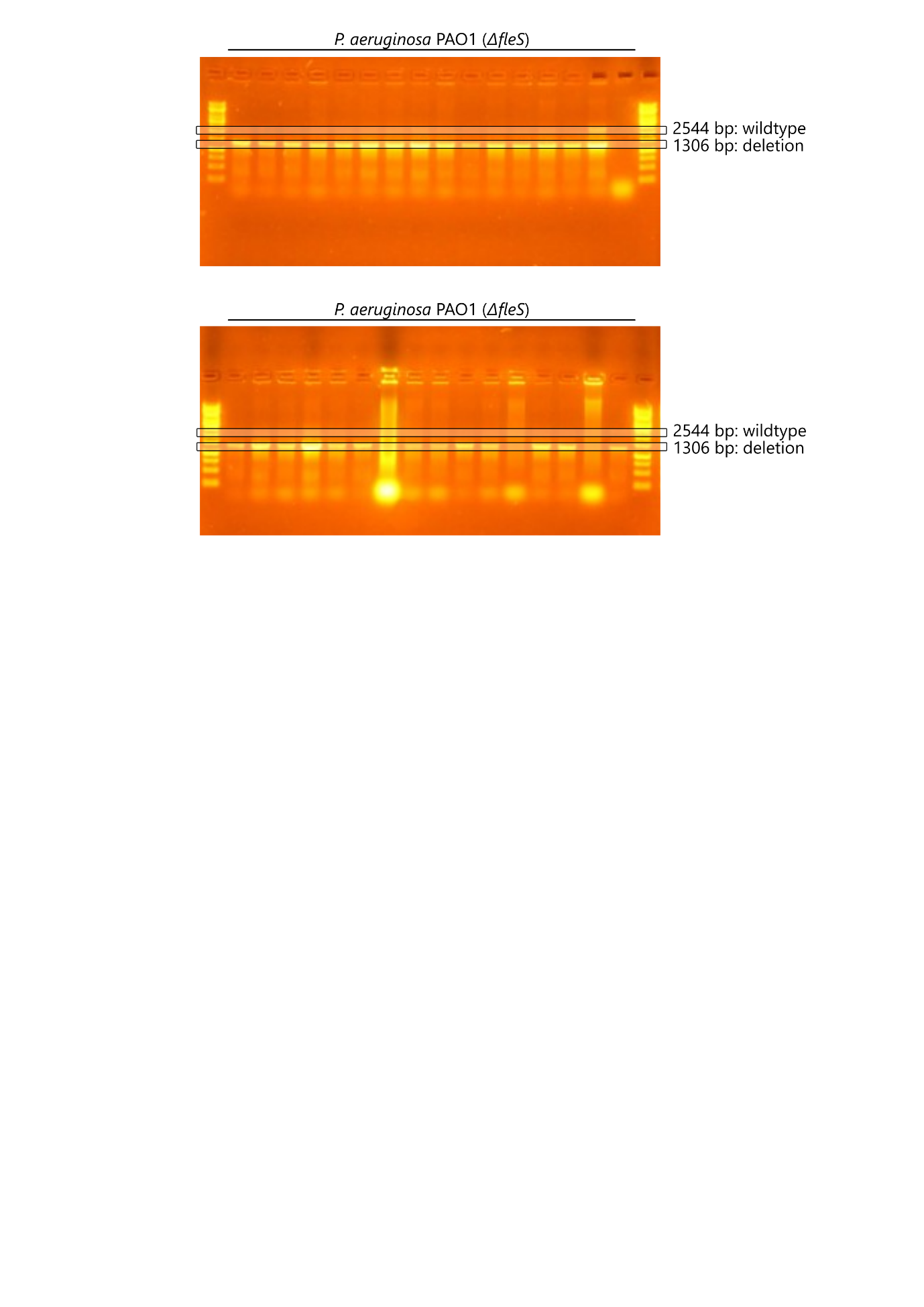


Figure S4: Deletion of fleS in P. aeruginosa PAO1 with Cas3-based engineering. A PCR screen was performed on co-transformants with genome-binding primers located outside of the homology arms. The first lane on each gel contains the commercial 1 kb GeneRuler (Thermo Scientific). Expected amplicon length of wildtype colonies (2544 bp) and deletion mutants (1306 bp) are indicated on each gel.


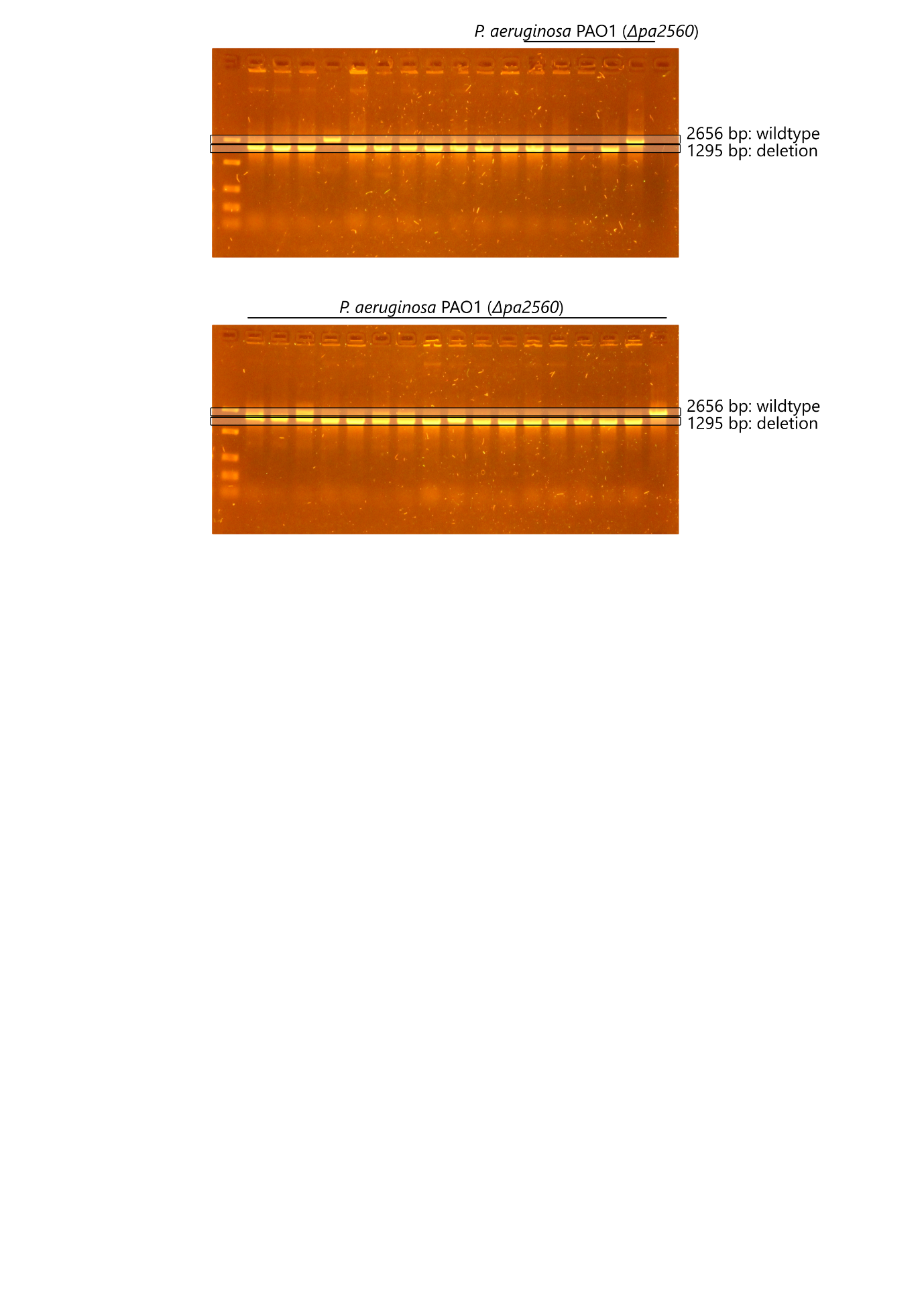


Figure S5: Deletion of PA_2560 in P. aeruginosa PAO1 with Cas3-based engineering. A PCR screen was performed on co-transformants with genome-binding primers located outside of the homology arms. The first lane on each gel contains the commercial FastRuler Low Range DNA ladder (Thermo Scientific). Expected amplicon length of wildtype colonies (2656 bp) and deletion mutants (1295 bp) are indicated on each gel.


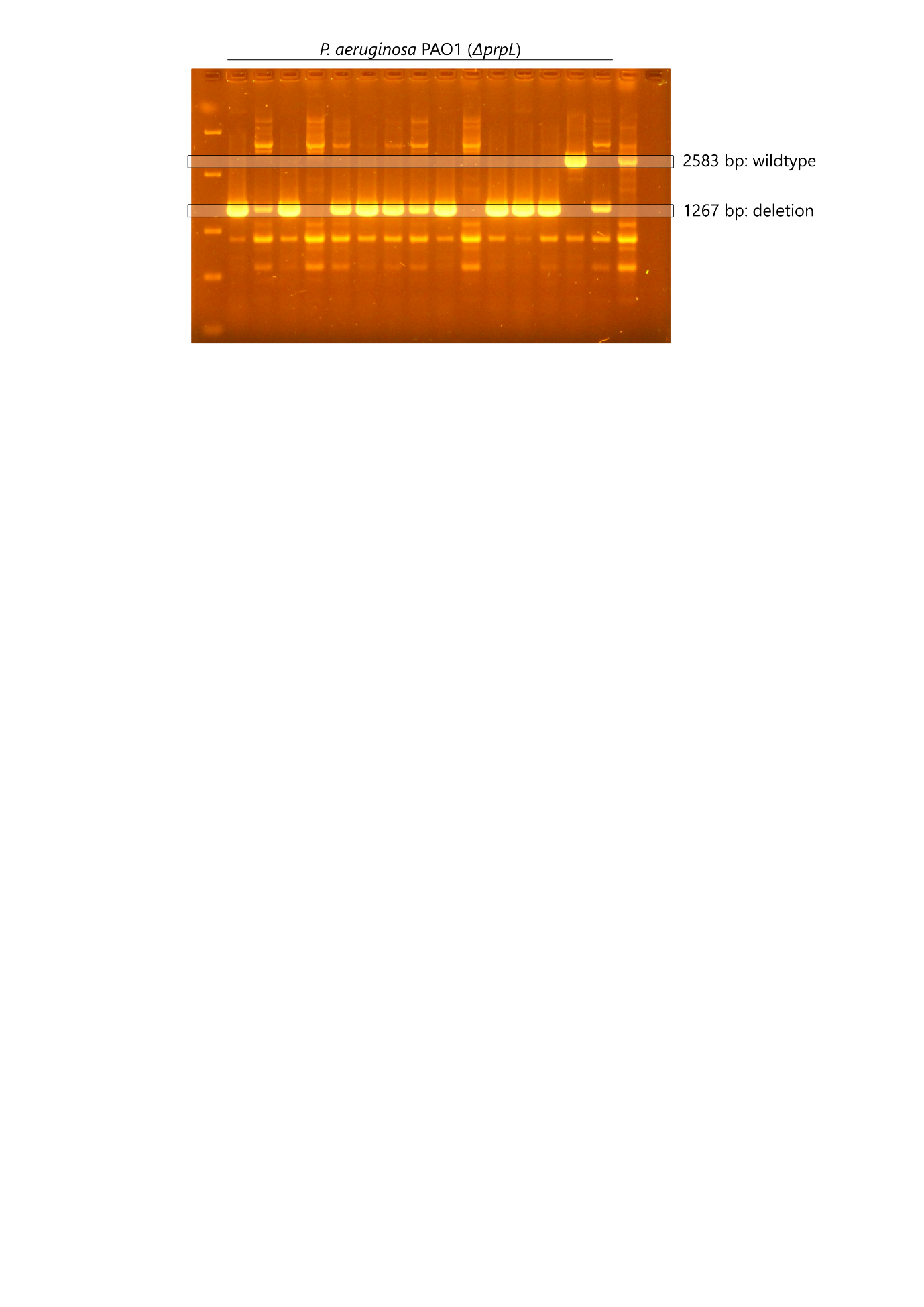


Figure S6: Deletion of prpL in P. aeruginosa PAO1 with Cas3-based engineering. A PCR screen was performed on co-transformants with genome-binding primers located outside of the homology arms. The first lane on each gel contains the commercial FastRuler Low Range DNA ladder (Thermo Scientific). Expected amplicon length of wildtype colonies (2583 bp) and deletion mutants (1267 bp) are indicated on each gel.


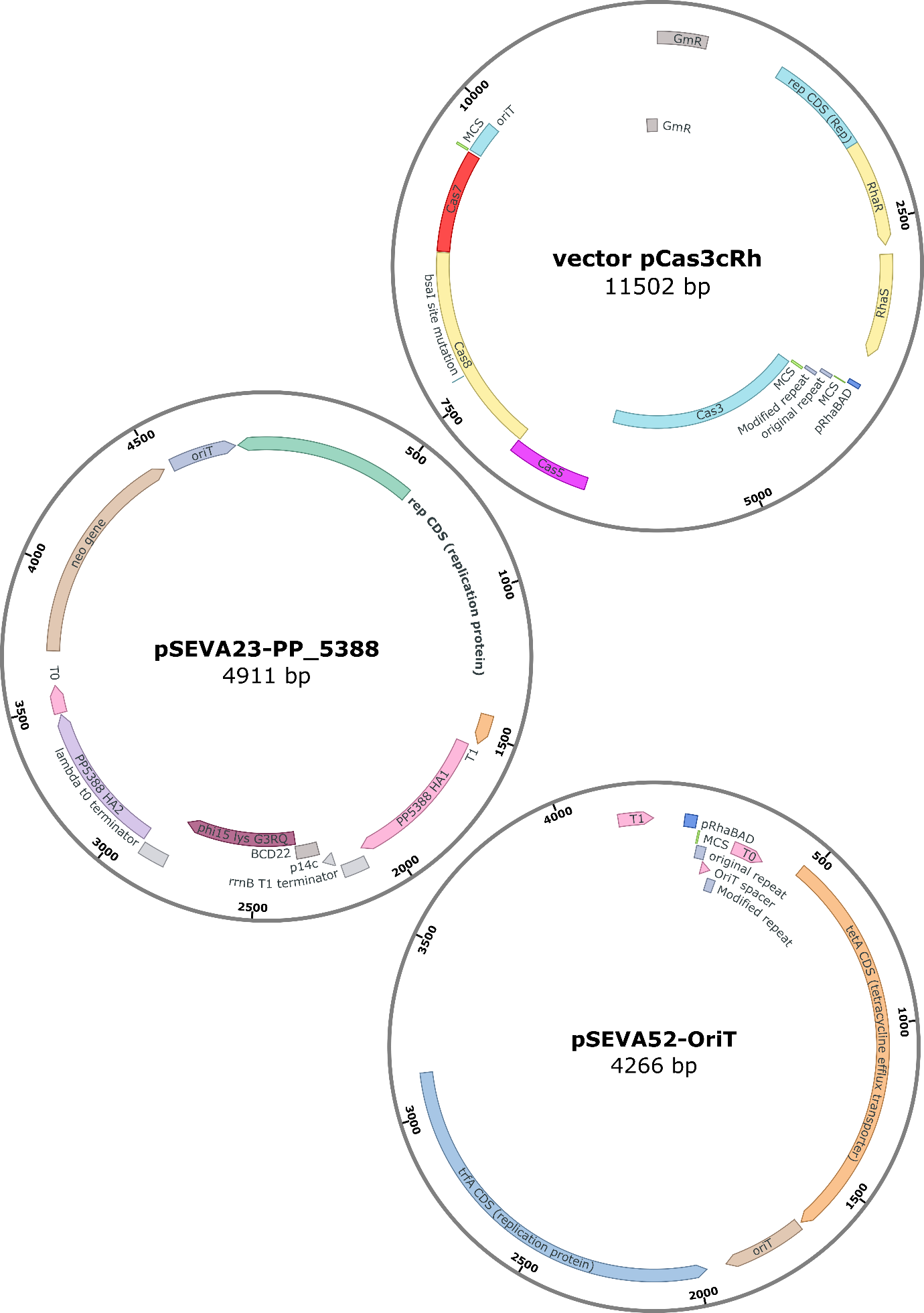


Figure S7: Vector maps of pCas3cRh, pSEVA23-PP_5388 and pSEVA52-OriT. CDS: coding sequence, ori: origin, T: terminator, p: promoter, BCD: bicistronic design, MCS: multiple cloning site

### Supplementary References

1. Csörgő B, León LM, Chau-Ly IJ, Vasquez-Rifo A, Berry JD, Mahendra C, Crawford ED, Lewis JD, Bondy-Denomy J. 2020. A compact Cascade–Cas3 system for targeted genome engineering. Nat Methods 17:1183–1190.

2. Silva-Rocha R, Martínez-García E, Calles B, Chavarría M, Arce-Rodríguez A, De Las Heras A, Páez-Espino AD, Durante-Rodríguez G, Kim J, Nikel PI, Platero R, De Lorenzo V. 2013. The Standard European Vector Architecture (SEVA): A coherent platform for the analysis and deployment of complex prokaryotic phenotypes. Nucleic Acids Res 41:666–675.

3. Volke DC, Wirth NT, Nikel PI. 2021. Rapid Genome Engineering of *Pseudomonas* Assisted by Fluorescent Markers and Tractable Curing of Plasmids. bio-protocol 11:1–16.

4. Zobel S, Benedetti I, Eisenbach L, De Lorenzo V, Wierckx N, Blank LM. 2015. Tn7-based device for calibrated heterologous gene expression in *Pseudomonas putida*. ACS Synth Biol 4:1341–1351.

5. Bagdasarian M, Lurz R, Rückert B, Franklin FCH, Bagdasarian MM, Frey J, Timmis KN. 1981. Specific-purpose plasmid cloning vectors II. Broad host range, high copy number, RSF 1010-derived vectors, and a host-vector system for gene cloning in *Pseudomonas*. Gene 16:237–247.

6. Wirth NT, Rohr K, Danchin A, Nikel PI. 2023. Recursive genome engineering decodes the evolutionary origin of an essential thymidylate kinase activity in *Pseudomonas putida* KT2440. MBio In press.

7. Stover CK, Pham XQ, Erwin AL, Mizoguchi SD, Warrener P, Hickey MJ, Brinkman FSL, Hufnagle WO, Kowallk DJ, Lagrou M, Garber RL, Goltry L, Tolentino E, Westbrock-Wadman S, Yuan Y, Brody LL, Coulter SN, Folger KR, Kas A, Larbig K, Lim R, Smith K, Spencer D, Wong GKS, Wu Z, Paulsen IT, Relzer J, Saler MH, Hancock REW, Lory S, Olson M V. 2000. Complete genome sequence of *Pseudomonas aeruginosa* PAO1, an opportunistic pathogen. Nature 406:959–964.
